# Scaling up community-delivered mental health support and care: A landscape analysis

**DOI:** 10.3389/fpubh.2022.992222

**Published:** 2022-12-08

**Authors:** Saher Siddiqui, Angelika Morris, Daniel J. Ikeda, Satchit Balsari, Laura Blanke, Miriam Pearsall, Roxanna Rodriguez, Shekhar Saxena, Benjamin F. Miller, Vikram Patel, John A. Naslund

**Affiliations:** ^1^Harvard University, Cambridge, MA, United States; ^2^Department of Global Health and Social Medicine, Harvard Medical School, Boston, MA, United States; ^3^Harvard Medical School, Boston, MA, United States; ^4^Department of Emergency Medicine, Beth Israel Deaconess Medical Center, Boston, MA, United States; ^5^Department of Global Health and Population, Harvard T.H. Chan School of Public Health, Boston, MA, United States; ^6^Well Being Trust, Oakland, CA, United States; ^7^Department of Psychiatry and Behavioral Sciences, School of Medicine, Stanford University, Stanford, CA, United States

**Keywords:** community initiated care, task sharing, community health worker, lay health worker, mental health, social determinants

## Abstract

**Introduction:**

The mental health crisis has caused widespread suffering and has been further exacerbated by the COVID-19 pandemic. Marginalized groups are especially affected, with many concerns rooted in social determinants of mental health. To stem this tide of suffering, consideration of approaches outside the traditional biomedical model will be necessary. Drawing from task-sharing models of mental health care that have been pioneered in low-resource settings, community-initiated care (CIC) represents a potentially promising collection of approaches. This landscape analysis seeks to identify examples of CIC that have been implemented outside of the research context, with the aim of identifying barriers and facilitators of scale up.

**Methods:**

A narrative review approach was used for this landscape analysis in which the PubMed database was searched and further supplemented with Google Scholar. Promising programs were then discussed over multiple rounds of meetings with the research team, consisting of collaborators with varied experiences in mental health. Using the selection criteria and feedback derived from group meetings, a final list of programs was identified and summarized according to common characteristics and features.

**Results:**

The initial PubMed search yielded 16 results, supplemented by review of the first 100 entries in Google Scholar. Through 5 follow-up meetings among team members, consensus was reached on a final list of 9 programs, which were grouped into three categories based on similar themes and topics: (1) approaches for the delivery of psychosocial interventions; (2) public health and integrative approaches to mental health; and (3) approaches for addressing youth mental health. Key facilitators to scale up included the importance of sustainable financing and human resources, addressing social determinants and stigma, engaging diverse stakeholders, leveraging existing health infrastructure, using sustainable training models, ensuring cultural relevance and appropriateness, and leveraging digital technologies.

**Discussion:**

This landscape analysis, though not an exhaustive summary of the literature, describes promising examples of efforts to scale up CIC outside of the research context. Going forward, it will be necessary to mobilize stakeholders at the community, health system, and government levels to effectively promote CIC.

## Introduction

Globally, the vast majority of individuals experiencing mental health problems lack access to adequate care, referred to broadly as the “care gap” (1). The COVID-19 pandemic has fueled a rise in the prevalence of mental health problems, disproportionately affecting young people, racial and ethnic minority groups, rural communities, and those who are economically disadvantaged (2). These are the same populations who are inequitably exposed to the social determinants of poor mental health, including economic stress, social isolation, and discrimination, and who may also have limited access to high-quality care (3). The traditional biomedical approach to mental health care, which places emphasis on a clinical diagnosis, use of pharmaceutical intervention, and reliance on mental health specialists as the first point of contact, will not be sufficient to stem the tide of suffering (1, 4). Business as usual has failed and governments and health systems are at an historic inflection point to reconsider and reimagine how population mental health can be addressed in more effective ways through broader structural reform.

Over the past two decades, efforts to leverage community resources have emerged as a key strategy to deliver evidence-based mental health interventions (5). A robust evidence base, now exceeding 100 randomized, controlled trials (6), demonstrates the acceptability, feasibility, and clinical effectiveness of the delivery of mental health care by non-specialist providers, such as community health workers, peers, lay people and nurses for a wide range of mental health problems in diverse contexts across the globe (7, 8). Successful interventions are anchored in the context of the communities into which they are deployed, and rely on sustained collaborations, funding, training, support, and monitoring (9). Commonly referred to as “task sharing” (7), this approach involves community-led identification and delivery of mental health care—in other words, community-initiated care (CIC). CIC involves building the capacity of local communities to implement evidence-informed programs to prevent and intervene upon mental health concerns (10). This approach represents a shift from the existing focus of mental health services on specialist-delivered care, by recognizing the key role that many caregivers and frontline workers can play in responding to the mental health needs in their communities. In some instances, care can even be initiated independent of the need for a formal diagnosis, where support can be provided at the community-level to individuals experiencing distress (5). This community-initiated approach to care holds potential to reach individuals who may be reluctant to seek help due to fear, stigma, costs, or mistrust of the formal health system—and could reduce persistent disparities in access to high-quality mental health care among under-resourced groups (4, 11). Empowering communities to become active partners in the delivery and implementation of mental health interventions could prevent worsening of early signs and symptoms of mental health problems (i.e., for persons with “sub-threshold” symptom severity), facilitate early intervention and linkage to formal care if needed, and ensure continuing support and follow-up care.

Drawing from the extensive global evidence base on task sharing, a 2021 report from the RAND Corporation makes a case for implementing CIC as part of a transformed mental health care system in the United States (12). Specifically, this report highlights three steps pertaining to CIC, including: (1) promoting pathways to care (e.g., promoting systematic mental health education); (2) improving access to care (e.g., implementing a national strategy to disseminate early interventions for serious mental illnesses); and (3) establishing an evidence-based continuum of care (e.g., launching a national care-coordination initiative) (12). This expands on other recent reports calling for the uptake of community-based initiatives to overcome global shortages in the mental health care workforce (13), scale up of community-based mental health and psychosocial support in humanitarian settings (14), and implementation of mental health task sharing in community-based organizations (15). Together, these reports reflect growing momentum toward recognizing the importance of task sharing and community-initiated efforts to address the global mental health care gap; however, it remains unclear whether there have been successful efforts to scale community-based initiatives beyond the research context.

The goal of this paper was to examine the landscape of CIC programs that have been implemented and scaled up beyond the research context. Specifically, our aim was to synthesize common barriers and enablers to implementation to subsequently inform the optimal design and scale up of CIC in other contexts.

## Methods

A narrative review approach was used for this landscape analysis to identify task-sharing and community-initiated care programs that have been scaled up in settings outside of the research context. For the purposes of this review, we defined “scaling up” according to the World Health Organization (WHO) definition, described as “deliberate efforts to increase the impact of health service innovations successfully tested in pilot or experimental projects so as to benefit more people and to foster policy and programme development on a lasting basis” (16). In line with this definition, scale up can entail increasing coverage of existing services, increasing the diversity of available services, or aligning existing services with new innovations in care delivery (16). A narrative review was considered appropriate because the literature on task sharing and other community-initiated approaches to mental health care has mainly comprised systematic reviews or meta-analyses summarizing randomized clinical trials (6, 8, 17). Few reports have considered the delivery of these programs beyond trial settings or research contexts. While there has been mounting recognition of the barriers and facilitators to implementation of mental health services, particularly involving use of task sharing in low-resource settings, recent reports have remained focused on research trials (18–20). Therefore, a narrative review approach was undertaken to identify the available evidence and determine the scope of program implementation beyond the research context, including key characteristics of these programs, and factors that may have enabled adoption and delivery.

### Eligible programs

Programs were selected according to the following criteria: (1) the program involves delivery in a community-based setting outside of the formal health care system (e.g., school, church, village); (2) the program involves use of an evidence-based intervention or is informed by scientific research to ensure that it is grounded in knowledge of what works for addressing mental health problems; and (3) the program has been implemented and scaled outside of the research context (i.e., beyond typical settings for clinical trials).

### Identifying the programs

The PubMed Medline database was searched on September 9, 2021, using the following terms: “community” AND “mental health” AND (“task sharing” OR “scaling up”). The focus was on identifying reviews published in English since January 1, 2015. We chose this year specifically to capture any reviews published since the search dates used in a prior major systematic review (8). Two members of the research team completed screening for reviews, and also screened the reference lists of these reviews to identify promising programs that had been evaluated and implemented. These two members had prior experience engaging in global mental health research and received additional training and supervision in landscape analysis methodology from the larger team throughout the search process (21). To supplement the database search, the first ten pages (~100 entries) of Google Scholar were searched using the same broad keywords (“scaling up,” “community,” “mental health,” and “task sharing”). Once programs were identified, we conducted a targeted search on Google to determine if there were any additional resources such as websites or descriptions of program implementation.

After this preliminary screening, acceptable articles were selected for inclusion in the landscape analysis through group discussion with all collaborators (included as study authors) until consensus was reached. All articles were reviewed in detail by all study authors prior to group discussion. This iterative review process consisted of five team meetings convened over five months. This collaborative approach promoted opportunities for the research team to resolve any disagreements and to reach consensus on the final programs for inclusion. The team composition was also important in guiding this review, as team members brought a range of experiences and expertise in research, clinical practice, policy, and advocacy work related to mental health.

### Data extraction and synthesis

Following selection of eligible programs, we began collecting relevant data from each program. We searched for additional information (e.g., websites, project reports, book chapters) to obtain data on programs that addressed the following questions:

- Where was the program delivered, in what country, and what type of community setting?
- Who delivered the intervention?
- Who are the beneficiaries of the program, and how were they identified and engaged in the program activities?
- What was the intervention delivered by these providers?
- To what extent has the program been scaled up in the target setting?
- What were the expected and observed outcomes or impact of the program at the individual and/or community level, and how were these assessed?

These key points were synthesized to outline strategies for guiding implementation of CIC in other settings.

## Results

The PubMed database search yielded a total of sixteen entries, supplemented by a screening of the first ten pages (~100 entries) in Google Scholar. After careful review and multiple rounds of discussion within the research team, a final list of nine programs was selected. These programs were grouped into three broad (and not mutually exclusive) categories based on similar themes and topics: (1) delivery of psychosocial interventions, (2) integrative public health approaches to mental health, and (3) programs addressing youth mental health. Details of each program are described in Table 1 and summarized in the sections below.

**Table 1:** Summary of selected programs.

**Name, location, references, funder**	**Type of provider**	**Focus population**	**Intervention**	**Evaluation**	**Scale-up success**	**Barriers/enablers**
***Category 1. Delivery of Psychosocial Therapies***						
The Friendship Bench, Zimbabwe (26–30) Multiple donors and partners	Lay health workers	People with mild to moderate common mental disorders	Trains lay health workers to provide CBT and problem-solving therapy	Compared to controls, participants (*n =* 286) had fewer symptoms on SSQ-14 (adjusted mean difference: −4.86, *p < * 0.001) and lower risk of symptoms of depression (13.7% vs. 49.9%, *p < * 0.001) (30)	Scaled up to 72 clinics across 3 cities in Zimbabwe; adapted for implementation in Malawi, Vietnam, United States	Barriers: difficulty measuring fidelity in scale up, program limited to adults Enablers: integrated within primary health care, clear referral pathways, locally validated tools
Thinking Healthy Program, originally in Pakistan (33–38, 40–46) Wellcome Trust	CHWs (original) and peers (following expansion)	Mothers with depression	Trains CHWs and peers to offer CBT integrated into maternal and child health education program	Relative to controls, participants (*n =* 349) had lower symptom severity on PHQ-9 (5.1 vs. 6.0, *p =* 0.003) and higher odds of remission (58% vs. 50%, *p =* 0.04) (37)	Recommended by WHO for treatment of perinatal depression; adapted for implementation in Bangladesh, China, Guatemala, Kenya, Peru, Vietnam	Barriers: Confidentiality concerns, stigma, financing peers Enablers: Coordination with district health offices, endorsement by WHO
StrongMinds, Uganda and Zambia (47–49) Multiple donors and partners	Mental health facilitators, local professionals trained in StrongMind's psychotherapy model	Women with depression	Trains mental health facilitators to provide IPT for 12 weeks of 60–90-min sessions	Over 80% of participants are depression-free, by PHQ-9, 6 months after the intervention; 16% report an increase in work attendance; 28% feel more socially connected (49)	Since 2014, StrongMinds has reached over 120,000 women and adolescents in Uganda and Zambia	Barriers: Stigma, low community awareness, skepticism about motives of program, dispersed local population Enablers: Community involvement, partnerships with other local organizations
***Category 2. Public Health and Integrative Approaches to Mental Health***						
Atmiyata, India (50, 52) Previously Grand Challenges Canada, now receives funding from Mariwala Health Institute with local NGO, Altruist, and TRIMBOS Institute	Local community volunteers from community resource groups, known as Champions	People with common mental health disorders and severe mental illness	Trains volunteers to detect, support, and refer people with common mental health disorders and severe mental illness	Relative to controls, individuals in the intervention (*n =* 829) had greater decreases in average GHQ-12 scores after the intervention (−0.16 vs. 0.69, *p =* 0.046) (52)	14,000 individuals have been screened, with 1,350 receiving mental health care and 1,350 receiving support with social benefits	Barriers: Low community awareness, skepticism about program motives, renumeration for Champions, difficulties using program app Enablers: supervision and support, peer-to-peer learning between Champions, community and stakeholder engagement
Fountain House, United States (53–58) Multiple donors and partners	Staff members and peers of Fountain House, a non-profit organization	Individuals with severe mental illness	Provides access to clinical support, housing, employment and education opportunities, and care management	Relative to propensity-matched controls, engagement in the program by participants (*n =* 285) was associated with a 21% reduction in Medicaid costs (55)	The model has been implemented in over 320 Clubhouses across 34 countries	Barriers: stigma, need for paid staff Enablers: wraparound services to address housing and employment
CONFESS, United States (59, 60) Multiple donors and partners	Barbers	Boys and men of color	Trains barbers in active listening, validation, feedback, and stigma reduction	No data available.	The program has certified over 600 barbers in 35 cities across 14 U.S. states	Barriers: stigma Enablers: community engagement, no renumeration of barbers
***Category 3. Youth Interventions—Education, Awareness, and Referral***						
Go-To Educator Program, Canada (65, 67, 69) Provincial health departments and local school boards	Teachers and other school staff	Students in classroom settings	Trains teachers to identify students with mental health needs and link them to care providers	Following participation, teachers reported improved scores in mental health knowledge (*n =* 920, 11.66 vs. 20.42, *p < * 0.001), and stigma (*n =* 873, 51.67 vs. 51.06, *p < * 0.001) (67)	An online version is currently under development with the goal of making the intervention more accessible	Barriers: challenges linking school and primary health systems, availability of core trainers Enablers: local champions to support adoption, flexible model
Going Off, Going Strong, Canada (70, 71) multiple donors and partners	Peers and community elders	Indigenous youth at risk of suicide	Focuses on providing social support and promoting transmission of traditional Intuit knowledge and skills	No data available	No data available	Barriers: lack of consistent funding, time- and resource-intensive model Enablers: community engagement, cultural relevance
headspace, Australia (73–77) Multiple donors and partners, including Australian government	Services are delivered via four main avenues: (1) headspace website, (2) eheadspace, (3) headspace centers (access to doctors, social workers, counselors, youth workers, etc.), (4) headspace school support	Young people aged 12–25 years	Addresses four key areas: (1) mental health and wellbeing, (2) physical and sexual health, (3) work, school, and study, (4) and alcohol and other drugs	Among students attending mental health services, 35.5% (*n =* 22,349) reported significant improvement in K10 scores (*p < * 0.001) and 36.1% (*n =* 24,997) reported significant improvement in SOFAS scores (*p < * 0.001) (75)	Since the program's inception, it has reached over 700,000 young people across Australia	Barriers: workforce capacity in rural settings Enablers: community engagement, family participation, on-site integration

CBT, cognitive behavioral therapy; CHW, community health worker; IPT, interpersonal therapy; PHQ-9, Patient Health Questionaire-9; SSQ-14, Shona Symptom Quesionnaire-14; GHQ-12, General Health Questionnaire; NGO, non-governmental organization; K10, Kessler Psychological Distress Scale; SOFAS, Social and Occupational Functioning Assessment Scale.

### Approaches for the delivery of psychosocial interventions

Psychosocial therapies are an effective approach for treating a range of mental illnesses (22, 23), and are recommended as a first-line treatment by the WHO (24). Despite strong evidence supporting the clinical effectiveness of these interventions in low-resource settings (6, 8, 17, 25), few programs have been implemented outside research contexts.

Originally developed in Zimbabwe, the Friendship Bench involves delivery of problem solving therapy (PST)–an approach for promoting individuals' self-efficacy and capacity to cope with common life stressors (26–30). As part of this model, community health workers are based in primary care clinics, and interactions with patients take place on benches within clinic premises. These health workers receive training in PST and behavior activation and are supervised by lay health workers known as “grandmother health providers” who have received training in cognitive behavioral therapy (CBT). The program consists of up to six structured, 45-min sessions, and offers access to support for patients throughout the care continuum. In a randomized, controlled trial of the program, patients who received the intervention had significantly fewer symptoms of depression as measured by the locally validated Shona Symptom Questionnaire (mean difference: −4.86, *p* < 0.001) (30, 31). The Friendship Bench has since been scaled up to over 70 clinics in the cities of Harare, Gweru, and Chitungwiza in Zimbabwe (27). The success in scaling up this program can largely be attributed to its integration within the healthcare system, where community health workers serve as a brige between informal and formal mental health care. Efforts are underway to adapt and deliver the Friendship Bench program in other countries and settings, including Malawi, Zanzibar, and New York City (32).

Like the Friendship Bench, the Thinking Healthy Program involves training community health workers, known as Lady Health Workers, to address mental disorders in low-resource settings using CBT principles and child education (33). Originally developed in Pakistan, the Thinking Healthy Program harnesses a tablet-based training and supervision platform to expand workforce capacity while enabling consistent supervision and skill-building across settings (34, 35). A cost comparison showed a 31% reduction in training costs (USD 117 vs. USD 170) when using a tablet-based platform compared to hands-on training by specialists (34). A pooled analysis of two cluster randomized controlled trials conducted in India and Pakistan found that participants who received the Thinking Healthy Program delivered by peers had lower symptom severity on PHQ-9 (5.1 vs. 6.0, *p* = 0.03) and higher odss of remission (i.e., PHQ-9 score < 5) (58% vs. 50%, *p* = 0.04) compared to a control group receiving usual care (36, 37).

Although the Thinking Healthy Program intervention was effective, a key barrier to scale up was identifying postpartum depression among women needing care (36). In many settings, postpartum depression carries significant stigma, which discourages disclosure and may contribute to under-reporting of symptoms (39). The Thinking Healthy Program has since been adopted by the WHO as part of its recommended interventions for psychosocial management of perinatal depression, and has been adapted for use in multiple countries, including China (40), Bangladesh (41), Vietnam (42), Peru (43), Guatemala (44), Kenya (45) and informal urban developments in Karachi, Pakistan (46). Key features that have enabled successful scale up include recognition from the WHO as a frontline treatment for perinatal depression, use of digital technology to facilitate health worker training, and use of a peer delivery model to increase sustainability and overcome challenges in workforce capacity.

With the same emphasis on addressing maternal depression, the StrongMinds program, delivered in Uganda and Zambia, utilizes lay community members to lead group interpersonal psychotherapy consisting of 60–90-min sessions delivered weekly over 12 weeks (47, 48). Since 2013, StrongMinds has reached more than 120,000 women and adolescents across Uganda and Zambia, with 86% of participants found to be depression-free by PHQ-9 at the end of treatment and sustained benefits observed at 6-month follow up (49). Furthermore, 28% of women reported feeling more socially connected, and there was a reported 30% increase in school attendance among children of participants (49). One of the key factors that helped to bolster the program's success was its emphasis on culturally relevant care. In addition, the program utilizes a train-the-trainer model, which ensures a consistent supply of human workforce capacity. This program is built on a model in which Mental Health Facilitators can recruit and train graduates of the program to become Peer Facilitators, who go on to lead their own therapy groups. The program's emphasis on forging partnerships between governments and non-governmental organizations has also enabled its successful scale up, allowing mental health services to be offered alongside existing programs that address food security, healthcare, and education. Finally, the program's focus on community engagement offers an opportunity to normalize conversations about mental health and reduce stigma.

### Public health and integrative approaches to mental health

Some programs have taken an integrative public health approach to mental health by bringing mental health services to locations outside of formal clinical spaces, and creating linkages to housing, employment, and education opportunities. Unlike traditional models that treat and support mental illness in clinical settings, these public health efforts seek to address mental health problems in a more holistic way by attending to social needs within community settings.

For example, the Atmiyata program has been implemented across multiple settings in India, and aims to raise awareness, support early detection, and provide support to people facing mental health challenges in rural communities (50, 51). Specifically, this program engages volunteers from established community groups and trains them to be community facilitators (referred to as Champions) and supporters (referred to as Mitras) (50). The week-long training of Champions is led by a psychiatrist, behavior change specialists, and Bharatiya Agro Industries Foundation Development Research Foundation employees, and consists of classroom-based lectures, films, interactive role plays, use of symptoms cards, and practice counseling sessions. Mitras receive one day of training, during which they are paired with Champions for support (50). A randomized, controlled trial of the Atmiyata program found that Champions were able to accurately identify people with emotional distress, and, after six counseling sessions, the proportion of participants with elevated symptoms dropped from 63.8 to 36.8% (52). While this program is based entirely within community settings, there is a mechanism through which lay health workers can refer individuals with more serious mental disorders to higher-level care (52). To ensure ongoing support and supervision of the Atmiyata Champions and Mitras, remote approaches utilizing mobile phones were combined with in-person supervision sessions (52). Leveraging technology to support supervision was essential for maintaining program fidelity and promoting successful scale up in a setting where supply of advanced clinical professionals is limited.

Another example of a successful program operating from an integrative public health perspective is Fountain House, based in New York City in the United States. Fountain House is the lead implementer of the Clubhouse model, which seeks to address mental health and social needs of members living with severe mental illness by providing access to peer support, case management, housing, employment, and education opportunities (53). Clubhouse is a model of psychosocial rehabilitation that leverages the power of peer support to promote recovery among those experiencing mental illness (54).

The success of Fountain House is reflected in a propensity score matching analysis which found that engagement in Fountain House programming was associated with a 21% reduction in Medicaid costs, although statistical power was notably limited due to small sample sizes (55). Fountain House has also experienced significant success in promoting housing and preventing recidivism among its members, reporting that 99% of members have stable housing within 1 year of enrollment (compared to 60% at enrollment) and only 5% of members with prior justice system involvement experience recidivism (56). The program also promotes paid, competitive employment of its members through vocational education and transitional and supported employment (54). Peer support plays a significant role across Fountain House programs and enables individuals with lived experience to provide support, share their own experiences, and facilitate recovery and rehabilitation among other members (57). Importantly, use of digital technology within Fountain House has played a key role in extending the reach and impact of its programs for members since the start of the COVID-19 pandemic (58). Globally, the Clubhouse model reaches over 100,000 people each year across more than 320 Clubhouses in 34 countries (54).

The CONFESS project also utilizes a peer support model to provide mental health promotion and support within barber shops to boys and men of color (59, 60). In some communities of color, barbers and hair stylists often play an informal counselor role that patrons may see as preferable to seeking formal mental health care in clinic or hospital settings (61–64). As part of the CONFESS project, barbers are trained in basic counseling skills and topics relevant to the mental health of their clients, including adverse childhood experiences, trauma, and suicide prevention. So far, the project's Beyond the Shop Program has trained more than 600 barbers in 35 cities across 14 states (59). Key facilitators of the CONFESS project's success include its leveraging of trusted community members, its use of a culturally tailored mental health curriculum, and its focus on peer support as an approach to mental health care.

### Approaches for addressing youth mental health

An additional group of programs focuses on education and mental health awareness, with a particular emphasis on youth. For example, in Canada, the Go-To Educator program involves training teachers and other school faculty to identify mental health concerns among students and link them to appropriate care (65). As teachers play a key role in supporting and mentoring their students, there is an important opportunity to integrate mental health education and awareness as part of this work (66). The Go-To Educator Program utilizes a train-the-trainer model consisting of a 2-day Core Trainer session that covers topics such as communication strategies and approaches to mental health promotion in schools (67, 68). These Core Trainers then proceed to train the “Go-To Educators” during a 1-day training session on youth mental health, stigma, triage, support, and referral (65). These Core Trainer groups continue to provide supervision to Go-To Educators as they take on their roles. An evaluation of the program using investigator-designed questionnaires of mental health knowledge and stigma found a significant improvement in knowledge of the epidemiology, social determinants, symptoms, and treatment of common mental disorders alongside significant reductions in negative attitudes toward individuals with mental disorders (67). The Go-To Educator program has been implemented in several provinces throughout Canada, including Nova Scotia, Ontario, Manitoba, and Alberta (67), with studies to evaluate program impact underway. For instance, the Alberta Mental Health Services in Calgary implemented the program within both public and Catholic school boards, and has trained over 1,600 teachers and school staff since 2016 (69).

The Going Off, Growing Strong program in Nunatsiavut in Northern Labrador, Canada, aims to reduce suicide risk among indigenous youth by promoting traditional vocational tasks and guidance from indigenous elders (70, 71). This community-based program focuses on promoting resilience among youth at elevated risk of suicide through linkages with traditional Inuit knowledge and skills (71). The Going Off, Growing Strong Program is particularly innovative in its recognition of intergenerational knowledge transmission as a mechanism for promoting resilience in indigenous communities (72), and its engagement of members of the indigenous community to lead the design and delivery of the program (71). Since the initial implementation of the program, anecdotal reports suggest that community suicide rates have decreased, with qualitative interviews revealing that youth feel more comfortable accessing help for negative thoughts, suicidal ideation, and chronic/traumatic stressors after participating in the program (70). Additionally, qualitative findings indicate that several participants obtained employment after gaining skills covered during the program (70).

Another program that focuses on adolescents is headspace (intentionally lowercase and not to be mistaken with the guided medication app, Headspace), which seeks to improve mental health outcomes through improved access to services among broad and diverse youth groups (ages 12 to 25) across Australia (73–76). This model focuses on early intervention and addresses mental, physical, and sexual health as well as alcohol and drug services and work and study support. The program provides a myriad of services including in-person support via headspace centers, online/phone support, work and study support, parents and carer events, an early psychosis program, and support for professionals and educators. Since its launch in 2006, headspace has reached over 700,000 young people in Australia (75). One study examining the services provided within headspace centers found that many young individuals came for mental health issues, as well as situational problems such as bullying (73). The wait time for appointments was 2 weeks or less for about 80% of clients, and only 5% waited more than 4 weeks (73). Although many of these services were provided by psychologists and allied mental health workers, the model also engaged peer intake workers and educators to provide counseling, support, and referral. Thus, while not entirely relying on task sharing and service delivery by lay providers, this model represents an important example of a hybrid model in which lay providers (i.e., peer supporters and schoolteachers) can serve as alternative entry points to receiving formal mental health care services. Another study sought to examine whether distance from headspace centers affected community awareness of headspace, and whether awareness of headspace centers changed between 2008 and 2015, finding that those who lived closer to headspace centers had significantly greater awareness of headspace and its services (74). In addition, awareness rose by 27% between 2008 and 2015. Headspace's annual report found that from 2020 to 2021 alone, 60,659 students participated across Australia (77). A key factor in the successful scale up of this program is the federal government's monetary support, symbolized by a pledge of nearly $300 million to the program to enhance the reach of headspace centers over the next 4 years (77).

## Discussion

This landscape analysis aimed to explore a selection of successful case examples to better understand how interventions that utilized the principles of CIC and task sharing were scaled up and sustained in non-research settings. From our literature review and multiple rounds of iterative discussions among team members, we identified nine promising case studies that were successfully scaled up. We summarized the characteristics of these programs and identified common themes and key features that were pertinent to either their successful delivery or difficulties in sustaining implementation. While these examples offer compelling evidence that approaches to CIC can expand the reach of services for supporting mental well-being at the community level, it is important to consider key barriers and enablers to achieving scale up and sustainment, including financing, human resource capacity, social determinants of mental health, stigma, stakeholder buy-in, and use of digital technologies.

### Financing

The community-based nature and simplicity of many task-sharing interventions allows care to be effectively delivered in resource-limited settings where mental health professionals are not readily available. When compared to traditional clinical care, these community-based approaches often require procuring external funding to sustain and scale. For more rural settings that may not have ample resources, it is often difficult to secure long-term funding for programming beyond initial time-limited research grants. While a program may be scalable through leveraging of local resources, ensuring sustainability represents a significant challenge. Among the programs summarized here, the Go-To Educator program was able to scale to many Canadian provinces; however, one barrier preventing it from achieving its full reach was human resources and funding constraints related to the mental health training and evaluation for the teachers (69). The program employs a train-the-trainer model to mitigate this challenge and is working to create an online version of the training so that it is more accessible to teachers who are interested in becoming involved (69).

### Human resource capacity

CIC and task sharing in general seek to alleviate the workforce burden by utilizing non-clinical support; yet, developing the supply side of a workforce remains a key challenge. Although lay workers, peer support specialists, and community members are more readily available as compared to mental health professionals, lay workers require a robust support infrastructure and ongoing training to ensure the success of the intervention and reduce the risk of staff turnover (78, 79). This challenge is relevant across many of the examples described here, including the CONFESS project (59), the Atmiyata program (50), and StrongMinds (49). Other programs draw from an existing workforce linked to the healthcare system in order to facilitate task sharing, as reflected in the Friendship Bench (26–30), the Thinking Healthy Program (33), and headspace (76), each of which offers important lessons for ensuring workforce sustainability. Specifically, the Friendship Bench is able to maintain its sustainability through existing health infrastructure and financing (26–30). Moreover, the use of existing lay health workers (rather than nurses), city health clinics, and supervisors employed by city health services was crucial to the sustained delivery of the Friendship Bench (29).

Similarly, the StrongMinds program in Uganda and Zambia successfully implemented a sustainable and self-replicating training model in which previous beneficiaries of the program train the next cohort of program beneficiaries (49). Yet, one limitation of this strategy is that peer facilitators, or previous beneficiaries of the program, may require additional supervision and support to ensure fidelity of program delivery. By integrating within existing health systems, these programs avoided the need to establish the clinical infrastructure necessary for reaching and referring patients, thereby allowing the programs to launch and achieve sustainability more easily. Furthermore, having the government engaged as a key partner can mobilize funding support as well as enable access to frontline health workers employed within existing health systems, which is critical to ensuring sustainability (27, 33, 75).

### Social determinants of mental health

Social determinants of mental health, which include factors such as economic stability, education access, and health care access, are known to impact individuals living with mental illnesses (80, 81). Models of CIC can make a concerted effort to address these issues from multidimensional perspectives, as exemplified in the Fountain House program. This approach requires consideration of not only members' mental health concerns, but also the social determinants of mental health to which their members are inequitably exposed (54).

### Stigma

Despite increasing awareness surrounding mental health at the community level, stigma continues to impede efforts to reach diverse population groups and can inhibit the implementation and scale up of mental health interventions (82). For instance, one of the challenges the Thinking Healthy Program faced was the identification of peers to support mothers experiencing depression (36), as this was hindered by the stigma attached to mothers experiencing postpartum depression (39). Supporting education and creating safe spaces where open discussions can take place can challenge misconceptions around mental health and help to reduce stigma (83). Consequently, for programs that seek to deliver psychosocial interventions to vulnerable populations, the integration of an educational component that focuses on challenging misconceptions about these illnesses is important for reducing stigma. Pertinent examples from this review include the Go-To Educator program (65) and CONFESS project (59), which included educational components to overcome misconceptions among individuals tasked with delivering the intervention.

### Multi-stakeholder buy-in

While many of the examples described in this review have demonstrated that political and health system buy-in are critical to success, it is also essential that the perspectives of both community leaders and other key stakeholders are considered. For example, the Friendship Bench identified political buy-in from key stakeholders, particularly the City Health Department and the Ministry of Health, as an essential component for scale up (29). On the other hand, the Going Off, Growing Strong program achieved success because the program included and related to community elders who offered mental health support and guidance (70, 71). However, one of the challenges the Go-To Educator program faced was promoting the intervention to schools and school boards, which could have potentially been alleviated through greater emphasis on engaging stakeholders within the school system and ministry of education (69). Ultimately, to maximize a program's chance at success and scalability, considering the perspectives of both political stakeholders, and local community members is crucial.

### Leveraging digital technology

When facing limitations on workforce and human resources, technology represent a common strategy utilized across many of these programs to transition components of the mental health interventions to online platforms. For example, the Thinking Healthy Program uses tablet-based training referred to as the “Technology-Assisted Cascade Training and Supervision System” to deliver the Thinking Healthy Program (34). Similarly, headspace leverages a prominent online presence, with support provided online and via phone, in combination with the use of in-person centers (77). Recently, the Go-To Educator program submitted a grant to develop an online version of its intervention to enable greater accessibility (69). However, the Going Off, Going Strong program has struggled with achieving full reach given the challenge of limited Internet access in its focus population. Thus in this circumstance, converting programming to an online format may not necessarily support scale up (70, 71). With the boom in use of digital technologies further accelerated by the COVID-19 pandemic, it is important to consider equity issues in terms of access, and specifically among populations that may not directly benefit from more information or mental health interventions being placed online (84).

### Summary

While the ability to secure private funding is crucial to maintaining a program's success, it was often those programs either supported by both local and federal governments which appeared to achieve the greatest success. Private donors or grant funding agencies cannot provide a limitless supply of financial resources, thus limiting the ability to scale and sustain many programs over the long term. This same mindset can be applied to human resources, where successful programs utilized lay workers that were supported through sustainable training programs or lay workers employed through the state, as observed in the Friendship Bench (26–30), Thinking Healthy Program (33), and StrongMinds (49). Additionally, acknowledging and further incorporating the social determinants of mental health into the program offerings was crucial to ensuring program impact, especially when serving marginalized populations. Some of the programs described here encountered stigma as a core challenge, which could be overcome through use of peer models of care (33, 49, 54, 59, 70, 71) as well as integrating educational components that directly challenge misconceptions about mental illness (65).

### Limitations

This landscape analysis has limitations. The overall paucity of published case studies on CIC and task sharing models implemented outside of the research context limited the scope of the analysis. While task sharing is widely described in the academic literature, it is primarily as part of clinical trials, with few descriptions of programs implemented outside of controlled research settings, and fewer yet being implemented in non-clinical contexts. The methodology of screening may have potentially limited the search results to only the most successful programs, failing to account for more recent and potentially informative programs that were not able to achieve scale or representation in the search results in this review. Similarly, although scale up has multiple dimensions (16), the relative lack of detail on scale up of programs precluded a more rigorous analysis of scale-up strategies. To address this limitation, our team engaged in multiple review meetings and open discussions, where informative programs not brought up in the initial search results were suggested and further investigated for consideration as part of the final analysis. Nonetheless, it is important to note that this search was not exhaustive, and there are likely other successful examples that have been implemented and scaled outside of research settings. Furthermore, our analysis only drew from studies that were described in English, which may have excluded successful case studies described in other languages.

Among the programs included in this landscape analysis, there were limitations with respect to the availability of information in the literature for each example. For instance, many of the programs did not have extensive information available on the support and training of providers or the mechanisms in place to ensure adequate support and quality assurance. In addition, there was insufficient information about the evaluation of some programs, making it difficult to extract metrics of success and impact. This potentially limits our understanding of the precise process that went into the pathway to scale for each of these programs. Similarly, many of the metrics of monitoring and evaluation, especially cost-effectiveness and cost-benefit analyses, were largely unavailable. Given the lack of these data, an important next step would be to hold workshops with stakeholders as part of the research process to identify these missing variables.

## Conclusion and implications for future research, policy, and practice

The programs summarized in this landscape analysis offer promising examples of efforts to scale up CIC and task sharing outside of the research context. This analysis sheds light on many essential enablers, which include integrating programs within existing workflows and infrastructure or integrating within community organizations that fall outside of formal health care settings, ensuring the availability of sufficient funding, and leveraging online tools and train-the-trainer methods to facilitate provider training and ongoing support. Alongside these valuable insights, however, lie several important challenges that require further consideration. For instance, such efforts will require tailoring programs to address the specific characteristics of the focus population including social determinants of mental health, culture, literacy, and access to technology. Similarly, the success of these programs will also require engaging and mobilizing stakeholders at the community, health system, and government levels to support adoption, overcome misconceptions about mental health, and sustain the implementation and rigorous evaluation of these programs. We sit at an important crossroads toward meaningfully addressing the mental health burden faced by communities across the globe. To realize this potential, continued advocacy and careful attention to the barriers and enablers to scale up and sustainment or program delivery will be essential.

## Author contributions

SSi and AM conducted the literature search and drafted the initial text with input from all authors. All authors participated in the iterative group discussions to reach consensus on the final selection of included studies, revised the work critically for important intellectual content, approved the final version to be published, and agreed to be accountable for all aspects of the work in ensuring that questions related to the accuracy of integrity of any part of the work are appropriately investigated and resolved.
